# EpCAM expression in squamous cell carcinoma of the uterine cervix detected by monoclonal antibody to the membrane-proximal part of EpCAM

**DOI:** 10.1186/s12885-017-3798-z

**Published:** 2017-12-04

**Authors:** Warangkana Chantima, Charin Thepthai, Pornsuk Cheunsuchon, Tararaj Dharakul

**Affiliations:** 1 Graduate Program in Immunology, Faculty of Medicine Siriraj Hospital, Mahidol University, Bangkok, Thailand; 2 Department of Immunology, Faculty of Medicine Siriraj Hospital, Mahidol University, Bangkok, Thailand; 3 Department of Pathology, Faculty of Medicine Siriraj Hospital, Mahidol University, Bangkok, Thailand

**Keywords:** Epithelial cell adhesion molecule (EpCAM), Membrane-proximal part, Membrane-distal part, Immunohistochemistry (IHC), Squamous cell carcinoma (SCC)

## Abstract

**Background:**

Epithelial cell adhesion molecule (EpCAM) is a promising biomarker for squamous cell carcinoma (SCC) of the uterine cervix, because it is over-expressed in various cancers of epithelial origin. However, EpCAM expression reported in previous immunohistochemistry (IHC) studies was inconsistent. We hypothesize that the membrane-distal part of EpCAM may be lost during tissue preparation, leaving only the membrane-proximal part of EpCAM available for antibody binding and IHC staining.

**Methods:**

Two new anti-EpCAM MAbs to the membrane-proximal part (WC-2) and the membrane-distal part (WC-1) of EpCAM were generated and characterized. WC-2 was selected for its ability to detect EpCAM in cervical tissues by IHC. One hundred thirty-five archival paraffin-embedded tissues previously diagnosed as cervical SCC (n=44), high-grade (HSIL) (n=43), or low-grade (LSIL) (n=48) squamous intraepithelial lesions were examined. IHC score was collected, recorded, and analyzed for distribution, intensity, and percentage of cancer cells stained for EpCAM.

**Results:**

EpCAM expression was consistently detected on cervical tissues by WC-2, but not by WC-1. EpCAM was expressed with high IHC score in the majority of cervical SCC (37/44), but not in normal epithelial area adjacent to SCC. EpCAM was also highly expressed on precancerous lesion of the cervix, particularly in HSIL. More importantly, EpCAM expression could be used to distinguish between HSIL and LSIL, according to staining distribution. HSIL tissues displayed EpCAM expression in two-thirds to full thickness of the epithelium, while in LSIL the staining was limited to the lower one-third of the thickness. The IHC score of EpCAM expression was strongly correlated with cervical cancer and grades of precancerous lesions (*r*=0.875, *p*<0.001).

**Conclusion:**

Only the anti-EpCAM MAb to the membrane-proximal part is able to detect EpCAM on paraffin-embedded cervical cancer tissues. A strong positive correlation between EpCAM expression level and the grades of SILs provides the possibility that EpCAM can be used to predict prognosis and severity in these patients.

## Background

Epithelial cell adhesion molecule is a cell-surface glycoprotein that is over-expressed in various cancers of epithelial origin. However, EpCAM expression investigated by immunohistochemistry (IHC) in squamous cell carcinoma (SCC) of the uterine cervix showed heterogeneity [1–6]. A study by Went, et al. [4] that used anti-EpCAM (VU-1D9) antibody reported EpCAM expression on only 17 of 42 SCC in paraffin embedded-tissues with moderate to high intensity. Using another anti-EpCAM (323/A3) antibody, Litvinov, et al. [5] reported EpCAM expression on cryostat sections of cervical SCC and cervical intraepithelial neoplasia (*n* = 15 and *n* = 39, respectively). EpCAM was occasionally detected at low intensity in the basal layer of normal ectocervical epithelia. However, the original publication that described 323/A3 antibody reported that EpCAM was not expressed on formalin-fixed, paraffin-embedded cervical SCC tissues [6]. Another 3 studies that used paraffin-embedded tissues reported that EpCAM expression was not detected on cervical SCC, although anti-EpCAM antibody clones were not mentioned in these studies [1–3]. Based on the aforementioned studies, we speculate that EpCAM expression is inconsistently detected on formalin-fixed, paraffin-embedded tissues, as compared to consistent detection observed in cryostat frozen sections. This difference in detection consistency may be due to the loss of the membrane-distal part of EpCAM molecule during tissue preparation for IHC.

**Fig. 1:**
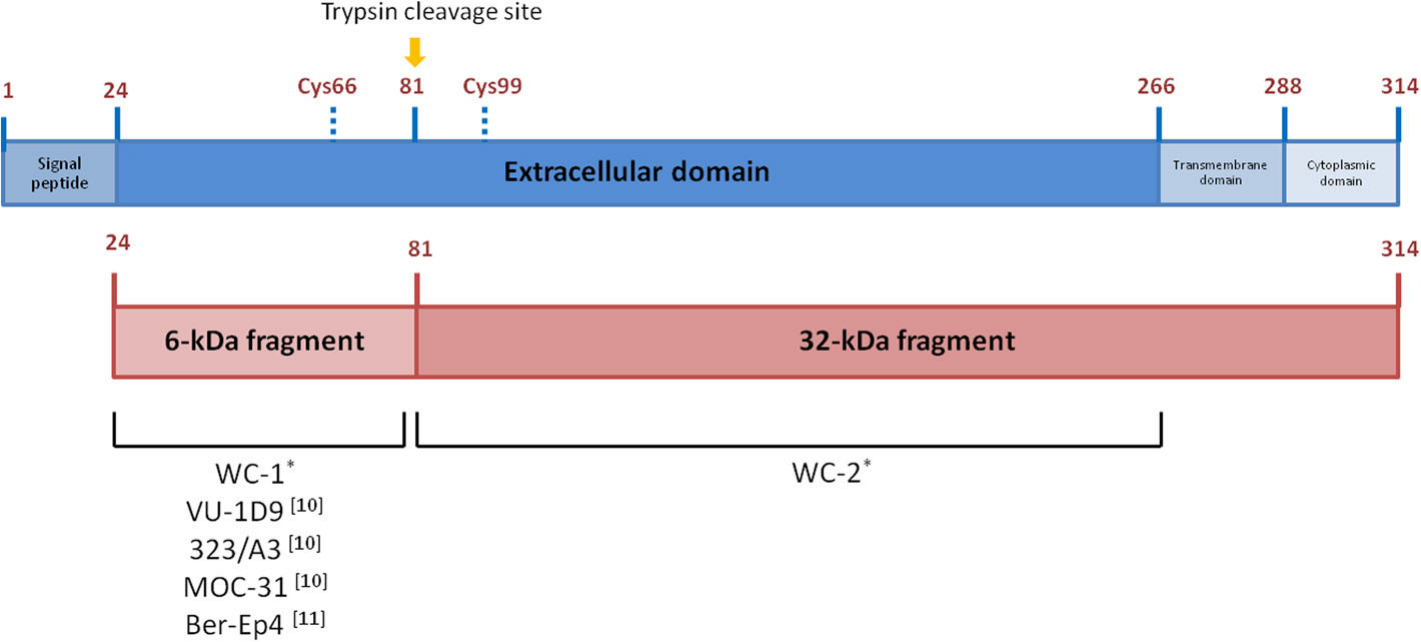
Diagram of EpCAM molecule presenting anti-EpCAM antibody recognition sites. 1–314: amino acid residue; cys: cysteine; WC-1, WC-2, VU-1D9, 323/A3, MOC-31, and Ber-Ep4: names of MAb; *MAbs produced in this study

When EpCAM is expressed on the surface of cancer cells, it can be cleaved by a variety of proteases, including trypsin, at the position Arginine80/Arginine81 (Fig. 1). This cleavage creates two fragments – 6 kDa and 32 kDa. The 6 kDa fragment is located distant from the cell membrane, whereas the 32 kDa fragment is located proximal to the membrane (Fig. 1). The fragments that are still held together by the disulfide bond between cysteine66 and cysteine99 after cleavage may be broken when exposed to reducing agents [7, 8]. During tissue preparation for IHC staining, the distal fragment of EpCAM can be removed by proteolytic enzymes, such as trypsin or pronase, during the antigen retrieval process. The anti-EpCAM antibodies used in previous studies, including VU-1D9 and 323/A3, recognized the 6 kDa fragment [6, 9, 10].

In this study, anti-EpCAM monoclonal antibodies that recognize the 6 kDa or the 32 kDa fragment of EpCAM extracellular domain were generated and compared to facilitate detection of EpCAM expression in cervical SCC. The antibody that exhibited intense IHC staining on cervical SCC with no background staining on normal tissue was then used to evaluate EpCAM as a biomarker on cancerous and precancerous lesions of the cervix.

## Methods

### Generation of anti-EpCAM monoclonal antibodies

Anti-EpCAM monoclonal antibodies were generated by hybridoma technique. The protocol for the mouse experiments was approved by Siriraj Animal Care and Use Committee (SiACUC), Faculty of Medicine Siriraj Hospital, Mahidol University (007/2554). BALB/c mice (National Laboratory Animal Center, Nakhon Pathom, Thailand) were immunized intraperitoneally with 50 μg of purified recombinant EpCAM protein corresponding to amino acid 24–266 of human EpCAM extracellular domain (Sino Biological, Inc., Beijing, P.R. China) in complete Freund’s adjuvant (Sigma-Aldrich, St. Louis, MO, USA). The mice were boosted with 50 μg of the same antigen in incomplete Freund’s adjuvant (Sigma-Aldrich, St. Louis, MO, USA) twice at a 4-week interval. A final boost with 50 μg of the same antigen in PBS by intravenous injection was performed 3 days before the mice were sacrificed. For hybridoma production, the spleen was collected aseptically and ground to separate spleen cells for fusion with myeloma cells P3-X63-Ag8.653. Hybridoma cells secreting anti-EpCAM antibodies were screened by ELISA using purified recombinant extracellular domain of human EpCAM as antigen. The hybridoma cells that secreted anti-EpCAM antibody were sub-cloned using limiting dilution method to obtain the monoclonal hybridomas. Monoclonal antibodies were then purified from cell culture supernatant using Protein G-Sepharose® affinity chromatography (GE Healthcare, Uppsala, Sweden).

### SDS-PAGE and western blot analysis

HT29 cells (EpCAM-positive cell line) were trypsinized and lysed in lysis buffer (1% triton X-100, 1 mM CaCl_2_, 50 mM Tris, pH 7.4). The lysate was then separated in 12% SDS-PAGE gel with or without 2-mercaptoethanol (reducing or non-reducing condition, respectively), and transferred to nitrocellulose membranes (Sartorious, Goettingen, Germany). The membrane was blocked and incubated with anti-EpCAM antibody at room temperature for 1 h. HRP-conjugated goat anti-mouse IgG (KPL, Inc. Gaithersburg, MD, USA) was used as secondary antibody. The signal was developed with ECL substrate (ThermoFisher Scientific, Waltham, MA, USA) and the image was captured by a Syngene gel documentation system (Syngene, Cambridge, UK).

### Tissue specimens

The protocol for this study was approved by Ethics Committee for Research in Humans, Faculty of Medicine Siriraj Hospital, Mahidol University (EC1260). One hundred and thirty-five archival paraffin-embedded tissues, previously diagnosed as cervical squamous cell carcinoma (SCC) (*n* = 44), high-grade (HSIL) (*n* = 43), or low-grade (LSIL) (*n* = 48) squamous intraepithelial lesions were included. Morphologically normal epithelia adjacent to the areas of SCC or SILs were identified in 54 tissues. All slides were stained by hematoxylin and eosin (H&E) and reviewed to confirm diagnosis.

### Immunohistochemistry (IHC) staining

The 5 μm thick paraffin-embedded tissue sections were cut onto poly-L-lysine-coated microscope slides (ThermoFisher Scientific, Waltham, MA, USA), deparaffinized, and rehydrated. Sections were incubated for 20 min at 95 °C with 0.01 M citrate buffer (pH 6.0) for antigen retrieval. Sections were stained using an autostainer (DAKO, Glostrup, Denmark) with 2 μg/ml of anti-EpCAM antibody for 30 min at RT. Sections were then incubated for 1 h at RT with HRP-conjugated goat anti-mouse IgG secondary antibody (DAKO, Glostrup, Denmark) and developed for 5 min with 3,3-diaminobenzidine (ThermoFisher Scientific, Waltham, MA, USA), followed by counterstaining using hematoxylin. Colon cancer tissue sections known to express EpCAM were used as a positive control in each batch of IHC staining.

### IHC scores

EpCAM expression was analyzed using 3 criteria, including staining distribution (A), staining intensity (B), and percentage of EpCAM positive cells (C). Staining distribution (A) was scored as: basal layer = 0, up to 1/3 of epithelium = 1, up to 2/3 of epithelium = 2, full thickness = 3. Staining intensity (B) was scored as: no staining = 0, weak intensity = 1, moderate intensity = 2, strong intensity = 3. Percentage of EpCAM positive cells (C) was scored as: 0–10% = 0, 11–40% = 1, 41–70% = 2, 71–100% = 3. Final IHC scores were calculated by the formula: IHC score = A × B × C. EpCAM expression levels were graded by IHC scores and divided into one of four groups, as follows: high (score = 18–27), medium (score = 6–12), low (score = 1–4) level, or no expression (score = 0).

### Statistical analysis

Predictive Analytics Software version 18.0 (SPSS, Inc., Chicago, IL, USA) was used for statistical analysis. Spearman correlation was used to analyze associations between IHC scores and grades of tissue abnormality.

## Results

### Anti-EpCAM WC-1 and WC-2 monoclonal antibodies and their recognition sites

The monoclonal antibodies directed against EpCAM extracellular domain were selected from a panel of monoclonal antibodies generated in our laboratory. The strategy for selection included ELISA using purified recombinant EpCAM extracellular domain. Sixteen hybridoma clones demonstrated strong reactivity with the recombinant EpCAM extracellular domain by ELISA. Two anti-EpCAM antibody clones, namely WC-1 and WC-2, were selected because they produced the strongest reactivity to the recombinant protein.

**Fig. 2:**
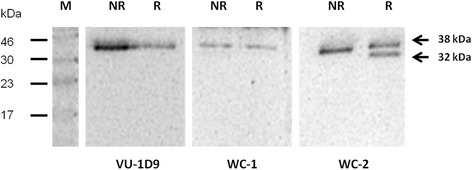
Western blot analysis of WC-1, WC-2, and VU-1D9 antibodies, showing that WC-2 recognized a 32 kDA proximal extracellular fragment of EpCAM molecule. Abbreviations: M, protein marker; NR, non-reducing condition; R, reducing condition

The recognition sites of WC-1 and WC-2 antibodies were analyzed by Western blot. The results showed that WC-2 recognized the 32 kDa and 38 kDa fragments under reducing condition, as shown in Fig. 2. The band of 38 kDa was still present because of the partial cleavage of EpCAM. In contrast, WC-1 antibody did not recognize the band of 32 kDa, but recognized the 38 kDa fragment. The commercial VU-1D9 anti-EpCAM antibody, which is known to recognize the epitope on 6 kDa fragment, showed the same pattern as WC-1.

### The ability of WC-1 and WC-2 to detect EpCAM in paraffin-embedded cervical cancer tissues by IHC staining

**Fig. 3:**
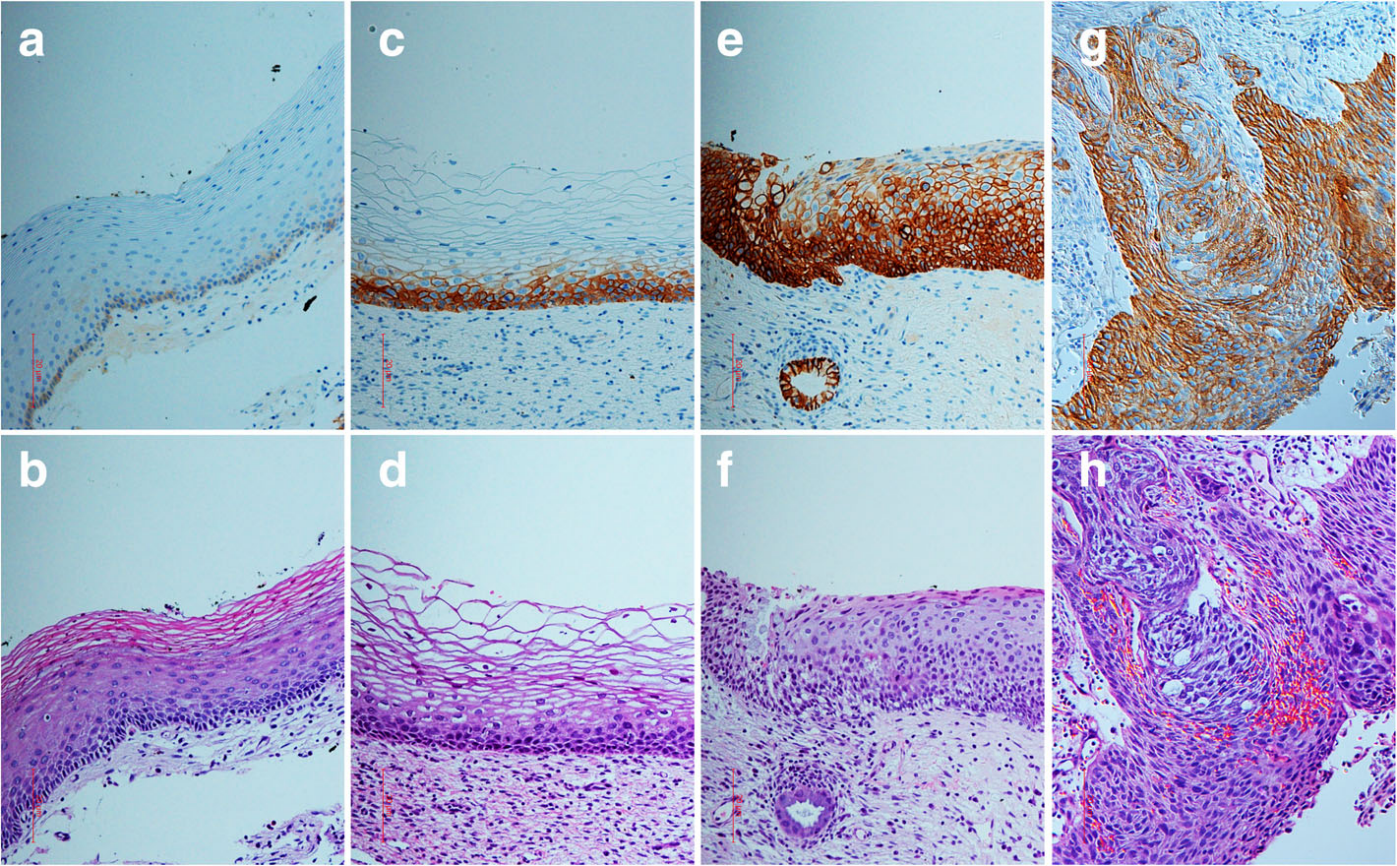
Immunohistochemistry staining of EpCAM using WC-2 antibody (**a**, **c**, **d**, **g**) and hematoxylin and eosin (H&E) staining (**b**, **d**, **f**, **h**) of ectocervical tissues. **a**, **b** normal ectocervical epithelium; **c**, **d** low-grade squamous intraepithelial lesion (LSIL); **e**, **f** high-grade squamous intraepithelial lesion (HSIL); **g**, **h** SCC of the uterine cervix

**Fig. 4:**
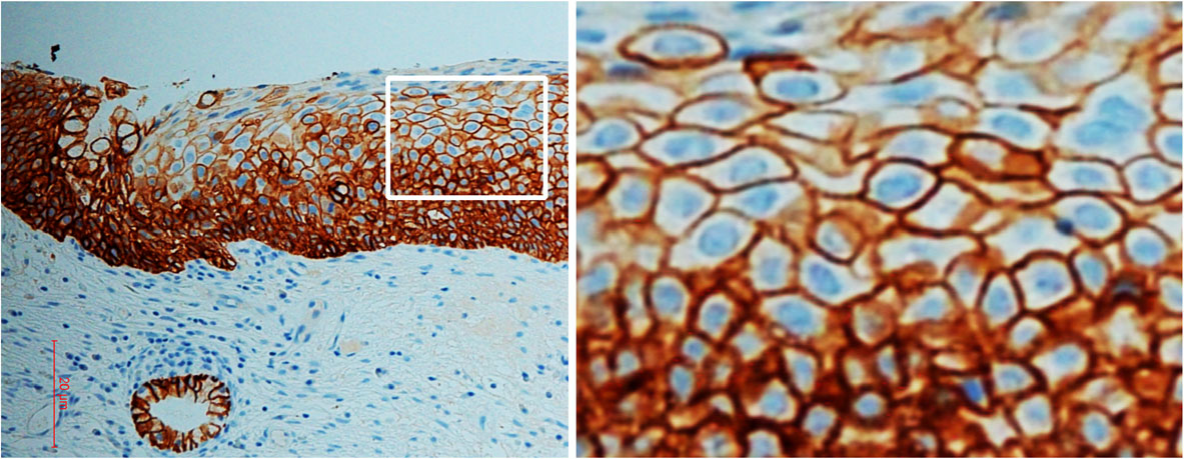
High-power magnification of Fig. 3e. EpCAM staining in abnormal squamous epithelium by WC-2 showed a cell-surface membranous staining pattern, and also a cytoplasmic pattern in some cells

Both WC-1 and WC-2 were further tested regarding their ability to detect EpCAM by IHC staining using paraffin-embedded cervical SCC tissue. Only WC-2 antibody exhibited particularly intense IHC staining on cervical SCC with no background staining on adjacent normal tissue (Fig. 3g-h, 3a-b, respectively). EpCAM staining by WC-2 demonstrated a cell-surface membranous staining pattern, and also a cytoplasmic pattern in some cells (as shown in Fig. 4 which represents a higher magnification of Fig. 3e).

In comparison with commercial anti-EpCAM antibodies, IHC staining using Ber-Ep4 or MOC-31, antibodies directed against 6 kDa fragment that are widely used for IHC [10, 11], were studied in 6 SCC tissue specimens that had intense EpCAM staining by WC-2. Some tissues had weakly positive staining with patchy distribution, in 1/6 and 4/6 by Ber-Ep4 and MOC-31 antibodies, respectively.

### EpCAM expression in cancer and precancer of the uterine cervix using WC-2

EpCAM expression on cervical tissues was analyzed in detail by IHC using WC-2 and was recorded in 3 categories, including staining distribution, staining intensity, and percentage of EpCAM positive cells (Table 1). The IHC staining pattern in SCC tissues demonstrated that, on the majority of SCC tissues (39/44), the full thickness of the epithelium was positive for EpCAM expression with moderate to intense intensity (Fig. 3g-h). In contrast, the ectocervical epithelium from noncancerous tissue or morphologically normal epithelium adjacent to the areas of SCC was negative for EpCAM. Weak staining was occasionally found on the basal layer (Fig. 3a-b).

**Table 1:** EpCAM expression pattern, including staining distribution, staining intensity, and percentage of EpCAM positive cells, in SCC, HSIL, and LSIL of the uterine cervix

	Specimen group								*r*	*p*-value
	SCC (*n* = 44)		HSIL (n = 43)		LSIL (n = 48)		Normal (*n* = 54)			
	n	(%)	n	(%)	n	(%)	n	(%)		
Staining distribution										
Full thickness	39	(88.6%)	29	(67.4%)	4	(8.3%)	0	(0%)	0.890	<0.001
Up to 2/3 of epithelium	5	(11.4%)	13	(30.2%)	10	(20.8%)	0	(0%)		
Up to 1/3 of epithelium	0	(0%)	1	(2.3%)	32	(66.7%)	4	(7.4%)		
Basal layer	0	(0%)	0	(0%)	1	(2.1%)	50	(92.6%)		
No staining	0	(0%)	0	(0%)	1	(2.1%)	0	(0%)		
Staining intensity										
Intense	29	(65.9%)	23	(53.5%)	0	(0%)	1	(1.9%)	0.698	<0.001
Moderate	14	(31.8%)	17	(39.5%)	29	(60.4%)	15	(27.8%)		
Weak	1	(2.3%)	3	(7.0%)	18	(37.5%)	38	(70.4%)		
No staining	0	(0%)	0	(0%)	1	(2.1%)	0	(0%)		
% of EpCAM positive cells										
71–100%	38	(86.4%)	38	(88.4%)	21	(43.8%)	21	(38.9%)	0.436	<0.001
41–70%	5	(11.4%)	5	(11.6%)	22	(45.8%)	25	(46.3%)		
11–40%	1	(2.3%)	0	(0%)	4	(8.3%)	8	(14.8%)		
0–10%	0	(0%)	0	(0%)	1	(2.1%)	0	(0%)		

*p*-value < 0.001 indicates statistical significance

EpCAM expression was also investigated on specimens with precancerous lesions of the uterine cervix, including high-grade (HSIL) and low-grade (LSIL) squamous intraepithelial lesions. In the majority of HSILs, EpCAM expression was positive up to two-thirds or full thickness of the epithelium with moderate to intense intensity (Fig. 3e-f). In LSILs, EpCAM was found on the lower one-third of the epithelium with weak to moderate intensity (Fig. 3c-d).

EpCAM expression levels were further graded into IHC scores and divided into groups as high (score = 18–27), medium (score = 6–12), low (score = 1–4), or no expression (score = 0) (Table 2). EpCAM expression level was high on the majority of cervical tissues with SCC (37/44) and HSIL (33/43), with low level (22/48) to medium level (21/48) expression observed on tissues with LSIL. EpCAM expression was not detected (50/54) or detected weakly (4/54) on normal cervical tissues. A strong positive correlation was found between levels of EpCAM expression and SCC, and grades of SILs (Spearman’s correlation *r* = 0.875, *p* < 0.001).

**Table 2:** Analysis of EpCAM expression levels in SCC, HSIL, and LSIL of the uterine cervix. Levels of EpCAM expression were graded by IHC scores

Specimen group	EpCAM expression								*r*	*p*-value
	High		Medium		Low		No expression			
	(IHC score = 18–27)		(IHC score = 6–12)		(IHC score = 1–4)		(IHC score = 0)			
	n	(%)	n	(%)	n	(%)	n	(%)		
SCC (n = 44)	37	(84.1%)	5	(11.4%)	2	(4.5%)	0	(0%)	0.875	<0.001
HSIL (n = 43)	33	(76.7%)	8	(18.6%)	2	(4.7%)	0	(0%)		
LSIL (n = 48)	3	(6.3%)	21	(43.8%)	22	(45.8%)	2	(4.2%)		
Normal (n = 54)	0	(0%)	0	(0%)	4	(7.4%)	50	(92.6%)		

*p*-value < 0.001 indicates statistical significance

## Discussion

In this study, we revealed that EpCAM expression can be consistently detected in paraffin-embedded cervical SCC tissues using a novel WC-2 antibody that recognized the 32 kDa fragment of EpCAM extracellular domain. In contrast, other anti-EpCAM antibodies that recognize the 6 kDa fragment cannot detect EpCAM expression. These findings support our hypothesis that the 32 kDa membrane-proximal fragment of EpCAM, but not the 6 kDa distal part, is preserved on paraffin-embedded tissues, and enables IHC staining of cervical cancer tissues. Anti-EpCAM antibodies against the 32 kDa fragment are more suitable for IHC staining. Although, a number of monoclonal antibodies to the 32 kDa fragment have been reported [9, 10, 12, 13], they were never used to study EpCAM expression in cervical cancer.

The present study strongly demonstrated that EpCAM expression was detected on the tissues of cervical SCC and precancerous SILs, and the expression levels correlated with grades of cell abnormality. Our results are in complete agreement with those of an earlier preliminary study by Litvinov, et al. [5] that studied the 323/A3 anti-EpCAM antibody using cryostat sections. We also found a statistically significant correlation between the levels of EpCAM expression and grades of cervical intraepithelial abnormality (correlation coefficient = 0.875, *p* < 0.001).

Recent studies have reported that several biomarkers can be used for cervical SCC, including Ki67 and p16^INK4A^ [14–16]. However, the expression of biomarkers is limited to the nucleus and cytoplasm of cervical cancer cells. To date, no cell-surface biomarker has been described for cervical SCC. The advantage of cell-surface biomarker is that it can be a target in immunodiagnostics or immunotherapy. For example, human epidermal growth factor receptor-2 (HER2/neu) is a target for antibody that exhibited therapeutic efficacy in metastatic and early-stage breast cancers [17, 18]. Moreover, human carcinoembryonic antigen (CEA) is a target in colorectal cancer immunotherapy [19, 20]. Here, we report that all cervical SCC tissues demonstrated EpCAM expression and this evidence supports that EpCAM can be a cell-surface biomarker for cervical SCC.

An important property of biomarker for cervical cancer is the ability to detect and differentiate between HSIL and LSIL at precancerous stages. This information can be used to aid in the diagnosis and in treatment planning. LSIL lesion has high regression rate, while HSIL has low rate of spontaneous regression and a substantial risk of developing into invasive cancer. Histologic differentiation between LSIL and HSIL is essential, as HSIL diagnosed on cervical biopsy requires excision or diagnostic excisional procedure, such as conization or loop electrosurgical excision procedure (LEEP) [21]. Analysis of our data indicated that EpCAM expression could distinguish between HSIL and LSIL by the staining distribution. HSIL tissues (42/43) displayed EpCAM expression in two-thirds to full thickness of the epithelium, while EpCAM staining in tissues with LSIL was limited to the lower one-third of epithelial thickness (34/48). Moreover, a strong positive correlation between EpCAM expression level and grades of SILs suggests the possibility that EpCAM can be used to predict prognosis and severity in patients.

## Conclusions

We report here for the first time that the membrane-proximal fragment of EpCAM extracellular domain is preserved on paraffin embedded-tissues and that it is required for IHC staining. This study also presents strong evidence that EpCAM expression is a suitable biomarker for cancer and precancerous lesions of the uterine cervix.

## Abbreviations

EpCAM: Epithelial Cell Adhesion Molecule
H&E: Hematoxylin and Eosin
HSIL: High-grade Squamous Intraepithelial Lesions
IHC: Immunohistochemistry
LSIL: Low-grade Squamous Intraepithelial Lesions
MAb: Monoclonal Antibody
SCC: Squamous Cell Carcinoma

## References

[1] Chao A, Wang TH, Lee YS, Hsueh S, Chao AS, Chang TC, Kung WH, Huang SL, Chao FY, Wei ML (2006). Molecular characterization of adenocarcinoma and squamous carcinoma of the uterine cervix using microarray analysis of gene expression. Int J Cancer.

[2] Imadome K, Iwakawa M, Nakawatari M, Fujita H, Kato S, Ohno T, Nakamura E, Ohkubo Y, Tamaki T, Kiyohara H (2010). Subtypes of cervical adenosquamous carcinomas classified by EpCAM expression related to radiosensitivity. Cancer Biol Ther.

[3] Spizzo G, Fong D, Wurm M, Ensinger C, Obrist P, Hofer C, Mazzoleni G, Gastl G, Went P (2011). EpCAM expression in primary tumour tissues and metastases: an immunohistochemical analysis. J Clin Pathol.

[4] Went PT, Lugli A, Meier S, Bundi M, Mirlacher M, Sauter G, Dirnhofer S (2004). Frequent EpCam protein expression in human carcinomas. Hum Pathol.

[5] Litvinov SV, van Driel W, van Rhijn CM, Bakker HA, van Krieken H, Fleuren GJ, Warnaar SO (1996). Expression of ep-CAM in cervical squamous epithelia correlates with an increased proliferation and the disappearance of markers for terminal differentiation. Am J Pathol.

[6] Edwards DP, Grzyb KT, Dressler LG, Mansel RE, Zava DT, Sledge GW, Jr., McGuire WL. Monoclonal antibody identification and characterization of a Mr 43,000 membrane glycoprotein associated with human breast cancer. Cancer Res 1986;46(3):1306–1317. 3510721

[7] Schnell U, Cirulli V, Giepmans BNG (2013). EpCAM. Structure and function in health and disease. Biochim Biophys Acta.

[8] Schnell U, Kuipers J, Giepmans BNG (2013). EpCAM proteolysis: new fragments with distinct functions?. Biosci Rep.

[9] Balzar M, Briaire-de Bruijn IH, Rees-Bakker HA, Prins FA, Helfrich W, de Leij L, Riethmuller G, Alberti S, Warnaar SO, Fleuren GJ (2001). Epidermal growth factor-like repeats mediate lateral and reciprocal interactions of ep-CAM molecules in homophilic adhesions. Mol Cell Biol.

[10] Balzar M, Winter MJ, de Boer CJ, Litvinov SV (1999). The biology of the 17-1A antigen (ep-CAM). J Mol Med (Berl).

[11] Winter MJ, Nagtegaal ID, van Krieken JH, Litvinov SV (2003). The epithelial cell adhesion molecule (ep-CAM) as a morphoregulatory molecule is a tool in surgical pathology. Am J Pathol.

[12] Helfrich W, Koning PW (1994). The TH, de Leij L. epitope mapping of SCLC-cluster-2 MAbs and generation of antibodies directed against new EGP-2 epitopes. Int J Cancer Suppl.

[13] Fornaro M, Dell'Arciprete R, Stella M, Bucci C, Nutini M, Capri MG, Alberti S (1995). Cloning of the gene encoding Trop-2, a cell-surface glycoprotein expressed by human carcinomas. Int J Cancer.

[14] Wentzensen N, Fetterman B, Castle PE, Schiffman M, Wood SN, Stiemerling E, Tokugawa D, Bodelon C, Poitras N, Lorey T et al. p16/Ki-67 Dual Stain Cytology for Detection of Cervical Precancer in HPV-Positive Women. J Natl Cancer Inst. 2015;107(12):djv257. 10.1093/jnci/djv257 PMC4675094 26376685

[15] Ikenberg H, Bergeron C, Schmidt D, Griesser H, Alameda F, Angeloni C, Bogers J, Dachez R, Denton K, Hariri J (2013). Screening for cervical cancer precursors with p16/Ki-67 dual-stained cytology: results of the PALMS study. J Natl Cancer Inst.

[16] Sahasrabuddhe VV, Luhn P, Wentzensen N (2011). Human papillomavirus and cervical cancer: biomarkers for improved prevention efforts. Future Microbiol.

[17] Chen JS, Lan K, Hung MC (2003). Strategies to target HER2/neu overexpression for cancer therapy. Drug Resist Updat.

[18] Emens LA (2005). Trastuzumab: targeted therapy for the management of HER-2/neu-overexpressing metastatic breast cancer. Am J Ther.

[19] Markman JL, Shiao SL (2015). Impact of the immune system and immunotherapy in colorectal cancer. J Gastrointest Oncol.

[20] Xiang B, Snook AE, Magee MS, Waldman SA (2014). Colorectal cancer immunotherapy. Discov Med.

[21] Massad LS, Einstein MH, Huh WK, Katki HA, Kinney WK, Schiffman M, Solomon D, Wentzensen N, Lawson HW (2013). 2012 updated consensus guidelines for the management of abnormal cervical cancer screening tests and cancer precursors. Obstet Gynecol.

